# Detailed analysis of electrogram peak frequency to guide ventricular tachycardia substrate mapping

**DOI:** 10.1093/europace/euae253

**Published:** 2024-09-29

**Authors:** Joseph Mayer, Jaffar Al-Sheikhli, Maria Niespialowska-Steuden, Ian Patchett, James Winter, Rafaella Siang, Nicolas Lellouche, Karthick Manoharan, Thanh Trung Phan, Justo Juliá Calvo, Andreu Porta-Sánchez, Ivo Roca-Luque, John Silberbauer, Tarvinder Dhanjal

**Affiliations:** Department of Cardiology, University Hospital Coventry and Warwickshire NHS Trust, CV2 2DX Coventry, UK; Department of Cardiology, Royal Stoke University Hospital, Stoke-on-Trent, UK; Department of Cardiology, University Hospital Coventry and Warwickshire NHS Trust, CV2 2DX Coventry, UK; Heart Rhythm Research Group, Division of Biomedical Sciences, Warwick Medical School, Clinical Sciences Research Laboratory, CV2 2DX Coventry, UK; Department of Cardiology, University Hospital Coventry and Warwickshire NHS Trust, CV2 2DX Coventry, UK; Department of Cardiology, University Hospital Coventry and Warwickshire NHS Trust, CV2 2DX Coventry, UK; Electrophysiology Division, Abbott Laboratories, Solihull, UK; Department of Cardiology, University Hospital Coventry and Warwickshire NHS Trust, CV2 2DX Coventry, UK; Heart Rhythm Research Group, Division of Biomedical Sciences, Warwick Medical School, Clinical Sciences Research Laboratory, CV2 2DX Coventry, UK; Department of Cardiology, Hopital Henri Mondor Albert Chenevier, Inserm U955, Paris, France; Sussex Cardiac Centre, Royal Sussex County Hospital, Brighton, UK; Department of Cardiology, Royal Stoke University Hospital, Stoke-on-Trent, UK; Sussex Cardiac Centre, Royal Sussex County Hospital, Brighton, UK; Arrhythmia Unit, Hospital Clínic de Barcelona, Barcelona, Spain; Arrhythmia Unit, Hospital Clínic de Barcelona, Barcelona, Spain; Sussex Cardiac Centre, Royal Sussex County Hospital, Brighton, UK; Department of Cardiology, University Hospital Coventry and Warwickshire NHS Trust, CV2 2DX Coventry, UK; Heart Rhythm Research Group, Division of Biomedical Sciences, Warwick Medical School, Clinical Sciences Research Laboratory, CV2 2DX Coventry, UK

**Keywords:** Ventricular tachycardia, Substrate mapping, Peak frequency, Amiodarone

## Abstract

**Aims:**

Differentiating near-field (NF) and far-field (FF) electrograms (EGMs) is crucial in identifying critical arrhythmogenic substrate during ventricular tachycardia (VT) ablation. A novel algorithm annotates NF-fractionated signals enabling EGM peak frequency (PF) determination using wavelet transformation. This study evaluated the algorithms’ effectiveness in identifying critical components of the VT circuit during substrate mapping.

**Methods and results:**

A multicentre, international cohort undergoing VT ablation was investigated. VT activation maps were used to demarcate the isthmus zone (IZ). Offline analysis was performed to evaluate the diagnostic performance of low-voltage area (LVA) PF substrate mapping. A total of 30 patients encompassing 198 935 EGMs were included. The IZ PF was significantly higher in sinus rhythm (SR) compared to right ventricular paced (RVp) substrate maps (234 Hz (195–294) vs. 197 Hz (166–220); *P* = 0.010). Compared to LVA PF, the IZ PF was significantly higher in both SR and RVp substrate maps (area under curve, AUC: 0.74 and 0.70, respectively). The LVA PF threshold of ≥200 Hz was optimal in SR maps (sensitivity 69%; specificity 64%) and RVp maps (sensitivity 60%; specificity 64%) in identifying the VT isthmus. In amiodarone-treated patients (*n* = 20), the SR substrate map IZ PF was significantly lower (222 Hz (186–257) vs. 303 Hz (244–375), *P* = 0.009) compared to amiodarone-naïve patients (*n* = 10). The ≥200 Hz LVA PF threshold resulted in an 80% freedom from VT with a trend towards reduced ablation lesions and radiofrequency times.

**Conclusion:**

LVA PF substrate mapping identifies critical components of the VT circuit with an optimal threshold of ≥200 Hz. Isthmus PF is influenced by chronic amiodarone therapy with lower values observed during RV pacing.

## Graphical Abstract

**Graphical Abstract:**
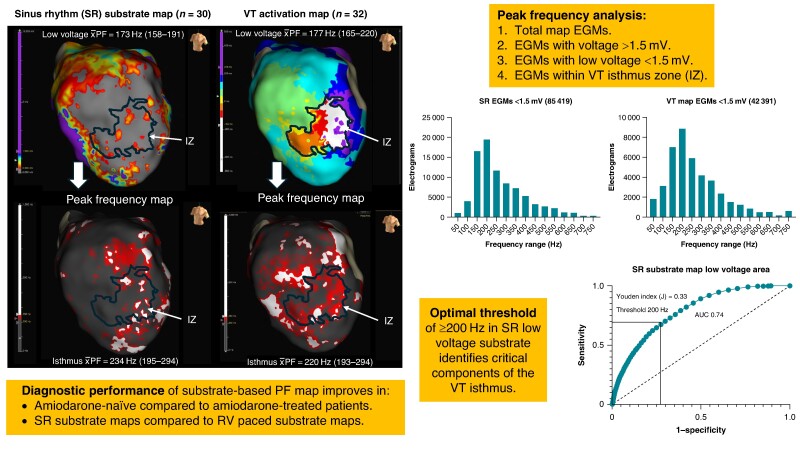

**What’s new?**

- Identification of critical components of the ventricular tachycardia isthmus is feasible using low-voltage–peak frequency substrate maps with an optimal peak frequency threshold of ≥200 Hz.
- The diagnostic performance of substrate-based, low-voltage–peak frequency mapping improves in amiodarone-naïve patients.
- Critical isthmus low-voltage–peak frequency values are lower during right ventricular pacing compared to sinus rhythm or during ventricular tachycardia.
- Low-voltage–peak frequency mapping to guide ventricular tachycardia ablation may improve the efficiency of the procedure.

## Introduction

Various substrate-based ablation approaches have been described for the identification of the critical components of the ventricular tachycardia (VT) circuit. These include core isolation, scar homogenization, de-channelling, as well as targeting late potentials (LPs), local abnormal ventricular activities (LAVAs), and ventricular electrogram duration mapping (VEDUM).^1–6^ There is increasing interest in refining the ablation target area with the selective targeting of functionally relevant substrate using techniques, such as isochronal late activation mapping (ILAM) and decrement-evoked potential (DeEP) mapping.^7–10^ The diagnostic performance of DeEP mapping has been shown to be greater than LP mapping in defining the critical VT isthmus.^9^ Compared to targeting LPs, the ILAM identification of deceleration zones has been shown to correlate better with successful ablation sites.^11^ Entrainment and VT activation mapping remain the gold standard approach in identifying the critical components of the VT circuit; however, these can be limited in clinical application due to haemodynamic instability and lack of inducibility secondary to antiarrhythmic drugs.

An important factor in identifying the arrhythmogenic low-voltage substrate beyond high-density mapping and algorithms that analyse different waveform directions is the ability to identify and differentiate between the near-field (NF) and far-field (FF) components of complex intracardiac electrograms (EGMs).^12–14^ The masking of relevant fractionated high-frequency components of the local EGM (NF) by adjacent higher-voltage myocardium (FF) can present challenges in identifying sites that are critical for the maintenance of VT. Indeed, this masking of relevant high-frequency substrate may be compounded by the use of chronic amiodarone therapy, which has been shown to prolong action potential duration, delay conduction velocity, and organise fractionated EGMs.^15–18^

A novel automated algorithm that annotates EGM signals using peak frequency (PF) during electroanatomic mapping (EAM) has been shown to accurately annotate NF-fractionated signals in the presence of FF components using wavelet transformation (WT).^19^ WT is a mathematical method that allows for frequency–domain analysis of a given signal, such as an EGM. Key to its utility in PF analysis is that WT provides information on both the frequency components of a signal and the timing of the frequency components (thus differentiating WT from the Fourier transform). Whereas Fourier transform-derived dominant frequency reﬂects the highest energy, the highest WT-derived PF is often associated with the lowest dominant frequency, as these EGMs are often low amplitude and highly fractionated. Using this approach, it is possible to derive the value and timing of the highest frequency within a given EGM. Importantly, whereas measures of the rate of change of voltage (dVdt) are influenced by signal amplitude, PF, as calculated by WT, is not. The WT algorithm can highlight areas of high frequency within a low-voltage substrate with ablation of high-frequency mid-diastolic potentials (MDPs) correlating with rapid VT termination.^20^ However, the diagnostic accuracy of substrate-based PF mapping in predicting the critical VT isthmus is unknown.

The aim of this study is to characterize the substrate-based PF maps and VT activation maps to^1^ evaluate the algorithms’ effectiveness in accurately identifying the critical mid-diastolic components of the VT circuit and^2^ determine the impact of chronic amiodarone therapy on PF-based mapping.

## Methods

### Study design and patient population

This is a multicentre, international cohort of adult patients with ischaemic and non-ischaemic cardiomyopathy undergoing VT ablation. Procedural indication included symptomatic VT despite optimised medical therapy, three or more episodes of VT requiring anti-tachycardia pacing (ATP), or at least one appropriate shock. The study complied with the Declaration of Helsinki. All patients provided written informed consent prior to the procedure with study approval provided by the University Hospital Coventry Research Department and the local research ethics committee. The data supporting the findings of the study are available from the corresponding author upon reasonable request.

### Procedural workflow

The ablation procedures were performed as previously described.^14,21^ Briefly, access to the LV endocardium was obtained via an antegrade transeptal or retrograde transaortic approach. Epicardial access was obtained using either the Sosa or Epi-CO_2_ techniques.^22^ Electroanatomical mapping was performed using the Advisor HD Grid Multipolar Mapping Catheter (Abbott, MN, USA) with the EnSite X Mapping System utilising the Omnipolar Technology Near-field (OTNF) Detection Algorithm (Abbott, MN, USA) using EGMs filtered 30–300 Hz.

Substrate maps were performed in sinus rhythm (SR) and/or RV paced (RVp) rhythm. Programmed ventricular stimulation (PVS) was performed to induce VT with the mapping catheter placed in the region of interest.^7^ Dependent on haemodynamic stability, a local activation timing (LAT) VT activation map was created to define the diastolic isthmus and terminate VT with radiofrequency (RF) ablation delivered using either the TactiFlex, TactiCath (Abbott, MN, USA), or DiamondTemp ablation catheter (Medtronic, MN, USA). Power settings for TactiFlex and TactiCath ablation catheters ranged from 40 to 50 W with a minimum force of 8 g aiming for an impedance drop of at least 10–15Ω.^4,8^ The DiamondTemp Catheter was temperature-controlled at 60°C with energy delivery, as previously described.^23,24^

Substrate elimination was performed as per institutional protocol, and operators were encouraged to ablate higher PF substrate utilising the OTNF algorithm with confirmation of substrate elimination with re-mapping. The post-ablation endpoint was VT non-inducibility with PVS.

### Retrospective off-line map and EGM analyses

All cases were reviewed retrospectively using the EnSite X Turbomap function to generate the SR substrate, RVp substrate, and VT activation maps using the OTNF algorithm. Criteria for case inclusion were strict with off-line map analysis if:

- there was full coverage of the low-voltage substrate using an interpolation distance of <7 mm within the SR substrate map;
- the VT activation map defined at least two-thirds of the diastolic isthmus with clear delineation between mid-diastolic potentials and lines of block; and
- there was full coverage of the low-voltage substrate using an interpolation distance of <7 mm within the RVp substrate map if RV pacing was performed.

**Figure 1:**
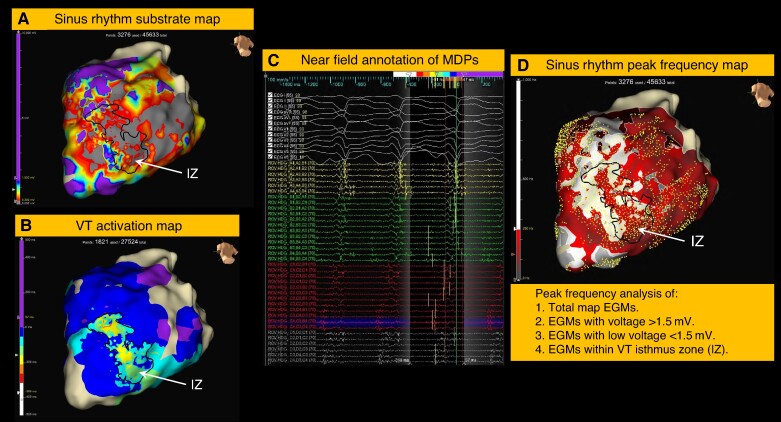
Study workflow to generate sinus rhythm, right ventricular paced, and VT activation maps for offline analysis. Using the Omnipolar Technology Near-field (OTNF) algorithm a SR substrate/voltage map (*A*) to identify the low-voltage area (LVA) (<1.5 mV) is followed by VT induction to generate the VT activation map (*B*) which identifies mid-diastolic potentials (MDPs) (*C*) and the isthmus zone (IZ). Vertical yellow lines identify the PF component of the complex EGMs used for timing annotation. The IZ has been demarcated in all maps (area within black line). The SR PF map is created with offline analysis of all EGM points (yellow squares) to determine the median PF within the total map area, viable voltage area (>1.5 mV), low voltage area (LVA) (<1.5 mV), and the IZ.

*Figure 1* summarises the methodological workflow for isthmus zone (IZ) characterisation and map analysis. The VT activation map was used to demarcate the diastolic isthmus with linear surface markers defining the boundary between mid-diastolic potentials and lines of block. In order to maintain an objective evaluation of the IZ within both the SR and RVp maps, the linear markers that defined the IZ (based on the VT activation map) were projected directly onto the same geometry of the SR and RVp maps (see Supplementary material online, *Figure S1*). The IZ criteria within the SR and RVp maps were therefore based on spatial coherence between the three map types.

The SR substrate, VT activation, and, if available, the RVp maps were then exported into a non-commercial bespoke offline analysis platform (Abbott, MN, USA). The low-voltage area (LVA) was defined as omnipolar voltage <1.5 mV to the noise floor (0.04 mV).^19^ The three maps were segmented offline to define: (a) healthy myocardium (>1.5 mV); (b) LVA scar region (1.5 to 0.04 mV); and (c) the IZ (see supplementary material online, *F*igure  *S1*). All map segments were examined for surface area, average omnipolar voltage, and median PF with values compared between the three map types and prespecified segments. Defining the IZ specifically within the LVA of the SR and RVp substrate maps enabled determination of the diagnostic accuracy (sensitivity and specificity) for the optimal LVA PF threshold in identifying the IZ using patient-wise vertically averaged receiver operating characteristic (ROC) curves.

### Optimal peak frequency threshold validation

Based on the prior analysis, the optimal LVA PF threshold was applied to the SR and RVp substrate maps using the electroanatomical emphasis map which highlights substrate within a prespecified frequency range. Maps were set to display high frequency using the optimal PF threshold. Two groups were identified: (i) cases with all ablation lesions located within 5 mm and (ii) cases with any ablation lesions located >5 mm of the optimal LVA PF map. Baseline characteristics, procedural data, and follow-up outcomes were compared between the two groups. All patients underwent a minimum of 6 months follow-up.

### Statistical analysis

For continuous variables with normal distribution, mean ± SD is reported. For those with non-normal distribution, median with 25 to 75% inter-quartile range (IQR) is expressed. The Shapiro–Wilk test was used for normality testing. For comparisons between the SR, RVp, and VT activation maps, analysis of variance was performed if data were normally distributed or Kruskal–Wallis test for non-normally distributed data. If a statistically significant global difference was found within the three maps, a *post hoc* comparison of continuous variables between the different maps was then performed using Student's *t*-test if data were normally distributed and Mann–Whitney *U* test with Bonferroni correction for non-normally distributed data. Categorical variables are reported as counts and proportions and displayed as percentages (%). Categorical variables were assessed using Fisher's exact test or χ^2^ test. Map comparisons were performed within the total cohort as well as within patient-matched maps. The ROC curve, AUC, sensitivity, and speciﬁcity analyses were performed to determine optimal PF thresholds. The Youden Index was used to deduce the optimal PF threshold at incremental frequency thresholds of 10 Hz (75 steps) to determine the best balance between the true positive rate and the false positive rate. GraphPad Prism version 10.0 (GraphPad Software, Boston, Massachusetts, USA) was used for statistical analysis.

## Results

### Study population and general procedural data

A total of 57 ablation cases were reviewed. Following exclusion for non-inducibility/incomplete VT activation maps and/or inadequate substrate coverage within the IZ and/or LVA, 30 patients were included in the final analysis. Baseline demographic and procedural characteristics are summarised in *Table 1*. The mean age was 64 ± 15 years, with an average ejection fraction of 34 ± 13%. Ischaemic cardiomyopathy was present in 90% of cases, and two-thirds of the cohort received chronic amiodarone therapy. Case spread included endocardial mapping from both ventricles as well as epicardial maps. Two VT activation maps were obtained from two patients, with the remaining patients presenting with one mappable VT. The average VT cycle length was 388 ± 87 ms, with a mean radiofrequency ablation time of 33 ± 20 min with 76 ± 42 ablation lesions. Post-ablation, 90% of patients were non-inducible, and 83% were free from any VT at 6 months.

**Table 1:** Baseline demographics and general procedural characteristics

Age at ablation, years	64 ± 15
Male sex, *n* (%)	25 (83)
Caucasian, *n* (%)	25 (83)
Aetiology	
Ischaemic cardiomyopathy, *n* (%)	27 (90)
Dilated cardiomyopathy, *n* (%)	1 (3)
Arrhythmogenic cardiomyopathy, *n* (%)	2 (7)
Clinical characteristics	
AF/flutter, *n* (%)	8 (27)
Hypertension, *n* (%)	17 (57)
Diabetes, *n* (%)	7 (23)
COPD, *n* (%)	5 (17)
Cerebrovascular disease, *n* (%)	5 (17)
CABG, *n* (%)	11 (36)
CKD, *n* (%)	11 (36)
Creatinine (µmol/L)	108 ± 35
LVEF (%)	34 ± 13
Previous VT ablation, *n* (%)	7 (23)
ICD, *n* (%)	26 (87)
CRTD, *n* (%)	4 (13)
Amiodarone, *n* (%)	20 (67)
Beta-blocker, *n* (%)	30 (100)
Mexiletine, *n* (%)	2 (7)
OAC/DAPT, *n* (%)	19 (63)
ICD shocks, *n* (%)	20 (67)
Presentation with VT storm, *n* (%)	7 (23)
**Procedural characteristics**	
General anaesthesia	9 (30)
Endocardial mapping	30 (100)
Epicardial mapping	2 (6)
LV mapping	30 (100)
RV mapping	3 (10)
Clinical VT cycle length (ms)	388 ± 87
Procedural time (mins)	275 ± 71
Ablation time (mins)	33 ± 20
Ablation lesions	76 ± 42
Post procedure VT non-inducibility	27 (90)
30-day complication	2 (6)
6-month freedom from any VT	25 (83)

**AF**, Atrial fibrillation; **COPD**, Chronic obstructive pulmonary disease; **CABG**, Coronary artery bypass grafts; **CKD**, Chronic kidney disease; **ICD**, Implantable cardioverter defibrillator; **CRTD**, Cardiac resynchronization therapy defibrillator; **LV**, Left ventricle; **RV**, Right ventricle; **LVEF**, Left ventricular ejection fraction; **OAC**, Oral anticoagulation; **DAPT**, dual anti-platelet therapy.

### Map characteristics and off-line peak frequency analysis

**Figure 2:**
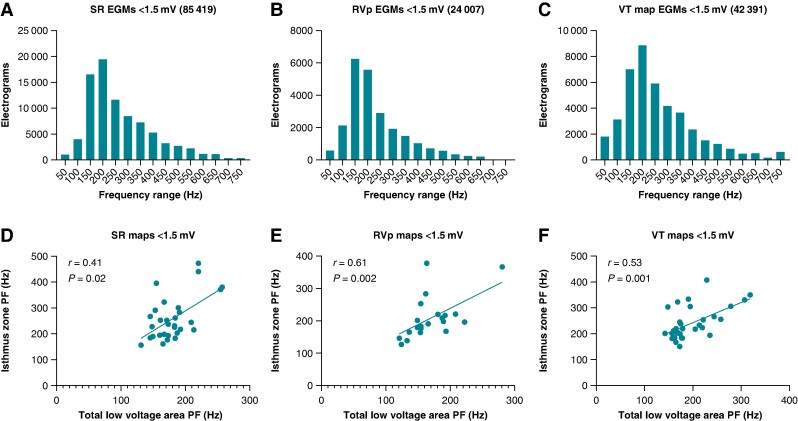
EGM peak frequency distribution graphs and scatter plot correlations within sinus rhythm, right ventricular paced, and VT activation maps. Low-voltage (<1.5 mV) area distribution graphs of PF in (*A*) SR substrate maps, (*B*) RV paced (RVp) substrate maps, and (*C*) VT activation maps. Corresponding scatter plots of VT isthmus area PF to low-voltage area PF are shown in D–F.

A total of 198 935 EGMs were analysed retrospectively off-line. This encompassed all EGMs formulating SR, RVp substrate maps, and VT activation maps. Of these EGMs, 151 817 were within the LVA (<1.5 mV), with all three map types demonstrating a negatively skewed distribution of PF, as shown in *Figure 2A–C*. Supplementary material online, *Figure S2* shows the negative skew of EGM PF within the entire maps and specifically within the IZ.

**Figure 3:**
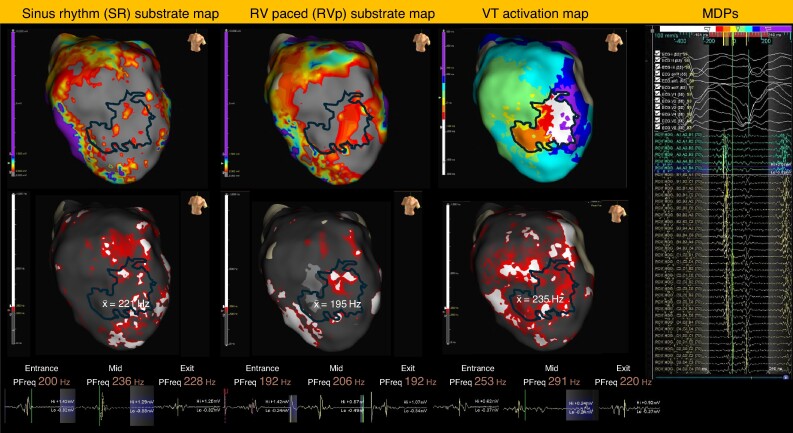
Graphical representation of offline map segmentation of the sinus rhythm, right ventricular paced, and VT activation maps to analyse peak frequency within low-voltage substrate and critical isthmus. SR substrate map, RVp substrate map, and VT activation map with IZ demarcated within black line with example OTNF-annotated MDPs. The corresponding PF maps are shown below (PF < 200 Hz = grey; PF 200–250 Hz = red; PF > 250 Hz = white), with the calculated average PF values within the IZ determined from 495, 275, and 361 EGMs, respectively. Note within the SR and RVp maps, the mid-isthmus region contains EGMs with PF values between 200 and 250 Hz. However, within the SR map, the EGM PFs are at the upper end of the 200–250 Hz range compared to the RVp map where EGM PFs are at the lower end of the 200–250 Hz range. Example EGMs with PF measurements are shown from each map at a similar location within the entrance, mid, and exit regions of the IZ. Vertical yellow bar indicates the PF potential within the complex EGM. Note high PF areas are highlighted in all three maps within the IZ, healthy myocardium, and septal apex near the RV pacing site.

Characteristics of the SR (*n* = 30), RVp (*n* = 21), and VT (*n* = 32) maps are outlined in Supplementary material online, *Table S1*. RVp substrate maps were not available for nine patients. A secondary analysis of 21 patients with all three map types is shown in Supplementary material online, *Table S2*. PF within the IZ varied significantly between the three map types (*P* = 0.025). There was no significant difference between SR and VT map IZ PF; however, the IZ PF was significantly lower in the RVp substrate maps compared to SR (*P* = 0.010) and VT (*P* = 0.022) maps. These observations were also present in the patient-matched map analysis (see Supplementary material online, *Table S2*). *Figure 3* highlights example EGMs recorded from similar locations within the demarcated IZ with differences in individual EGM PF and average IZ PF between the three map types.

### SR and RVp low-voltage area peak frequency and co-localisation to the VT isthmus

**Figure 4:**
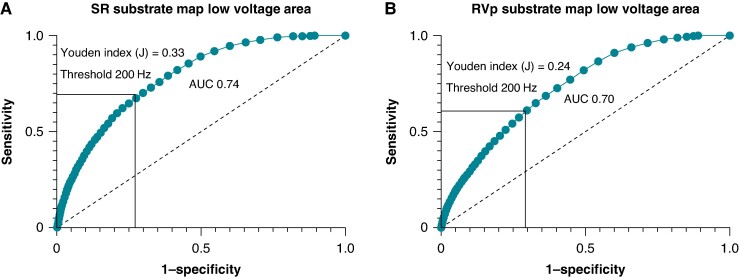
Receiver operating characteristic (ROC) curves to determine optimal peak frequency thresholds. ROCs of PF within low-voltage area and PF within the IZ in (*A*) SR substrate maps and (*B*) RV paced substrate maps.

The diagnostic performance of SR and RVp LVA substrate PF in identifying the IZ was assessed. A moderate correlation was observed between LVA PF and IZ PF within all three map types ,as shown in *Figure 2*(*D–F*). *Table 2* shows a comparison of the median PF value from the SR and RVp map LVA compared to the median PF value within the IZ from the corresponding map type. Significant increases in IZ PF compared to LVA PF were observed in both the SR and RVp substrate maps with AUCs of 0.74 and 0.70, respectively. The ROC curve analysis, as shown in *Figure 4*, revealed an optimal PF threshold of ≥200 Hz for the SR LVA substrate PF in identifying the IZ, with a sensitivity of 69% and a specificity of 64%. With the RVp LVA substrate map, the sensitivity was 60% with a specificity of 64%.

**Table 2:** Comparison of low-voltage (<1.5 mV) substrate peak frequency to the VT isthmus zone peak frequency

	Low-voltage area PF, Hz (IQR)	VT IZ PF, Hz (IQR)	% PF increase	*P*-value	AUC
SR map	173 (158–191)	234 (195–294)	36%	**<0.001**	0.74
RVp map	162 (148–190)	197 (166–220)	22%	**<0**.**001**	0.70

**SR**: Sinus rhythm; **RVp**: Right ventricular paced; **VT**: Ventricular tachycardia; **IZ:** Isthmus zone; **IQR:** Interquartile range; **PF:** Peak frequency; **Hz:** Hertz; **AUC**: Area under curve.

### Impact of chronic amiodarone therapy on peak frequency

A total of 20 patients received chronic amiodarone therapy. The influence of amiodarone on map characteristics compared to amiodarone-naïve patients is displayed in *Table 3*. Within the LVA, the PF was significantly lower within the IZ of VT activation maps in amiodarone-treated patients [172 Hz (163–178) vs. 229 Hz (190–257), *P* = 0.002]. Importantly, within the IZ of SR substrate maps, PF was significantly lower in amiodarone-treated patients [222 Hz (186–257) vs. 303 Hz (244–375), *P* = 0.009]. Two amiodarone-treated patients were additionally taking mexiletine. The ROC analysis demonstrated a higher AUC during both SR (AUC: 0.77 v 0.74) and RVp mapping (0.74 v 0.71) in amiodarone-naïve patients. The optimal PF threshold for amiodarone-naïve SR LVA substrate maps was ≥220 Hz to identify the VT isthmus, with a sensitivity of 73% and a specificity of 67% (J = 0.40). The optimal PF threshold for amiodarone-naïve RVp LVA substrate maps was ≥200 Hz to identify the VT isthmus, with a sensitivity of 65% and a specificity of 66% (J = 0.31).

**Table 3:** Comparison of amiodarone-treated vs. amiodarone-naïve cohorts and map characteristics

	Sinus rhythm substrate map			Right ventricular paced substrate map			VT activation map		
	Amiodarone	No Amiodarone	*P*-value	Amiodarone	No Amiodarone	*P*-value	Amiodarone	No Amiodarone	*P*-value
Age, yrs	67 ± 16	59 ± 14	0.05	67 ± 18	54 ± 13	**0.02**	67 ± 16	59 ± 14	0.05
LVEF (%)	35 ± 13	31 ± 12	0.34	33 ± 11	29 ± 10	0.42	35 ± 13	30 ± 12	0.34
ICM, *n* (%)	17 (85)	10 (100)	0.53	13 (93)	5 (71)	0.55	17 (85)	10 (100)	0.53
Number of maps	20	10		14	7		21	11	
Total map PF, Hz (IQR)	194 (184–209)	214 (200–250)	0.54	175 (162–198)	199 (192–227)	0.09	193 (173–214)	236 (209–257)	**0**.**003**
>1.5 mV area PF, Hz (IQR)	260 (221–288)	286 (254–306)	0.11	232 (211–280)	251 (233–306)	0.28	229 (216–288)	249 (223–291)	0.76
Low voltage (<1.5 mV) area PF, Hz (IQR)	172 (154–186)	182 (159–228)	0.21	154 (144–188)	186 (162–191)	0.17	172 (163–178)	229 (190–257)	**0**.**002**
VT IZ PF, Hz (IQR)	222 (186–257)	303 (244–375)	**0**.**009**	190 (160–212)	213 (198–329)	0.07	212 (188–247)	256 (194–331)	0.09

**IQR**, Interquartile range; **IZ**, Isthmus zone; **LVEF**, Left ventricular ejection fraction; **ICM**, Ischaemic cardiomyopathy; **VT**, Ventricular tachycardia; **PF**, Peak frequency; **Hz**, Hertz; **mV**, Millivolts.

### Validation of ≥200 Hz peak frequency threshold

**Figure 5:**
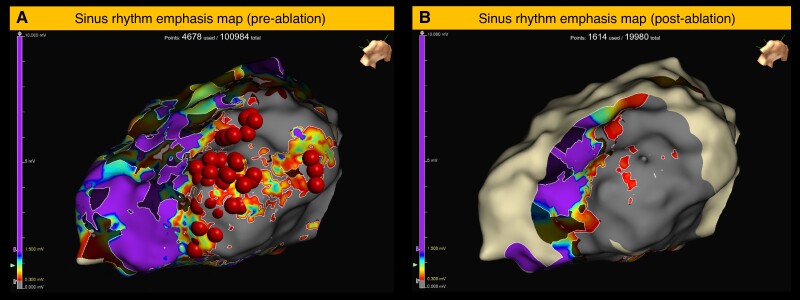
Graphical representation of example validation case with emphasis maps pre- and post-ablation. SR substrate map with PF ≥ 200 Hz is emphasised. The automated lesion tag algorithm (Autotag, Abbott, MN, USA) was used to visualise ablation lesions with manual verification of lesion delivery sites in all maps. All ablation lesions are within 5 mm of the low voltage-high PF region. Post-ablation re-map shows a near complete elimination of low-voltage–high-PF region.

The ≥200 Hz PF threshold to identify relevant low-voltage substrate in either SR or RVp maps was applied to the retrospective cohort. In 15 patients, all ablation lesions were within 5 mm of the threshold applied map, as shown in *Figure 5A*. *Table 4* shows the differences between the patients who received catheter ablation with all ablation lesions within 5 mm of the ≥200 Hz PF threshold (*n* = 15) compared to those with any ablation lesions located >5 mm of the ≥200 Hz PF threshold (*n* = 15). The baseline characteristics were comparable with no significant differences in acute VT non-inducibility or 6-month freedom from any VT; however, in the ≥200 Hz PF cohort, there was a trend towards reduced ablation lesions and RF times. Six patients underwent re-mapping following ablation with sufficient coverage of the IZ. *Figure 5* highlights an example case of emphasis maps pre- and post-ablation. Within the remapped cases, there was a reduction in IZ PF of 155 Hz (61–248) (*P* = 0.004).

**Table 4:** Analysis of differences between those who received catheter ablation with all lesions <5 mm of the ≥200 hz PF threshold substrate map compared to patients with ablation lesions located >5 mm from optimal PF threshold substrate map

	All ablation lesions <5 mm of the optimal threshold map (*n* = 15)	Ablation lesions >5 mm of the optimal threshold map (*n* = 15)	*P*-value
Age, yrs	64 ± 16	64 ± 15	0.97
LVEF (%)	36 ± 12	31 ± 13	0.30
ICM, *n* (%)	14 (93)	13 (86)	>0.99
Clinical VT cycle length (ms)	394 ± 88	383 ± 89	0.79
Ablation time (mins)	29 ± 17	38 ± 39	0.36
No of ablation lesions	62 ± 41	89 ± 39	0.05
Post-procedure VT inducibility, *n* (%)	0	3 (20)	0.22
30-Day complication, *n* (%)	1 (6.6)	1 (6.6)	>0.99
6-Month freedom from any VT, *n* (%)	12 (80)	13 (87)	>0.99

**LVEF**, Left ventricular ejection fraction; **ICM**, Ischaemic cardiomyopathy; **PF**, Peak Frequency; **Hz**, Hertz; **mV**, Millivolts.

## Discussion

The present study provides a comprehensive insight into PF mapping during VT ablation. The major findings of this study are as follows:

1. identification of critical components of the VT isthmus is feasible using substrate-based PF maps to highlight relevant low voltage with an optimal PF threshold of ≥200 Hz;
2. the diagnostic performance of substrate-based PF maps improves in amiodarone-naïve patients; and
3. low-voltage substrate-based PF mapping during SR may identify the critical VT isthmus more accurately than RV pacing.

There are several reasons why PF map analysis was focused within low-voltage substrate. Firstly, viable ‘healthy’ myocardium displayed high-frequency ranges similar to the isthmus region. Secondly, conventional mapping strategies are focused on chamber region of interest guided by electrocardiographic, voltage, and/or imaging data. Finally, the practical implementation of the emphasis map feature that combines both frequency and voltage enables a frequency threshold to be displayed across a voltage range.

The present study highlights an optimal threshold of ≥200 Hz within low-voltage areas in co-localising the IZ. This is comparable to the recent findings from Payne et al., where an optimal threshold of 216.5 Hz correlated with manually identified LPs and LAVAs.^19^ This study also reported similar PF values in the overall scar area and significantly higher PF values in areas that may represent potential ablation targets. A cut-off of 220 Hz was shown to correlate with areas of ILAM.^19^ An important distinction in the diagnostic accuracy testing of PF within the present study is the correlation directly to the critical VT isthmus, as opposed to LPs and LAVAs. Furthermore, bespoke offline analysis enabled PF analysis of all map EGMs, reducing the risk of bias through manual verification.

### Diagnostic accuracy of mapping techniques

Multiple mapping methodologies have been reported to define critical sites. The relationship of critical sites to low-voltage areas has been previously reported with 84% of entrance and mid-isthmus sites localized to dense scar and 68% of critical sites localized to border-zone substrate.^25,26^ Identification of low-voltage channels has a poor specificity (30%) for the VT isthmus and is limited by automated annotation to the FF EGM component, which is frequently higher in voltage than the NF potential of interest.^27^ As a result, voltage criteria alone lack specificity for isthmus sites.^26,28^ The present study findings show that combining PF analysis with low-voltage substrate improves the specificity to the VT isthmus.

Functional substrate identification techniques, such as ILAM and DeEP mapping, have emerged to refine the mapping process with improved accuracy in defining critical isthmus sites. ILAM-defined DZs have been shown to correlate to 95% of VT termination sites.^7^ DeEP mapping in pre-defined areas of interest has a specificity of 97% with a sensitivity of 61%.^9^ As in the present study, the diagnostic accuracy testing was based on the location of the diastolic isthmus determined from the VT activation map. Using an optimised DeEP protocol, we have previously shown in whole-chamber DeEP mapping that the specificity of DeEPs to detect the VT isthmus was 83%, with a sensitivity of 85%.^8^ With a single sensed extra from the RV apex to induce decrement, a specificity of 96% and a sensitivity of 87% were achieved; however, diagnostic performance was based on best entrainment and pacemap sites as opposed to identification of the IZ.^29^ LP maps to identify critical isthmus sites have reported specificities ranging from 65 to 82% and sensitivities ranging from 60 to 78%.^9,29^ Interestingly, DeEP mapping has been shown to be influenced by activation wavefront direction.^30^ The present study also highlights that localised isthmus PF values are affected by RV pacing compared to SR maps and during VT. The IZ PF was significantly greater (*P* = 0.01) in the SR substrate maps (234 Hz) compared to the RV paced substrate maps (197 Hz).

Payne et al. showed a sensitivity of 91% and a specificity of 85% for a PF cut-off of 220 Hz in low-voltage fractionated areas to correlate with manually verified LAVAs and LPs.^19^ In the present study, we report a specificity of 64% and a sensitivity of 69% with SR substrate maps and a specificity of 64% and sensitivity of 60% with RV paced substrate maps to identify the critical diastolic isthmus. On balance, the diagnostic accuracy of low-voltage PF mapping is better than voltage-based approaches alone; however, it may not perform as well as functional substrate mapping techniques.

### Impact of chronic amiodarone therapy on peak frequency

Amiodarone non-competitively blocks both alpha- and beta-adrenergic receptors and increases action potential duration prolonging repolarisation and refractoriness.^15^ The antiarrhythmic action persists up to 45 days following discontinuation of drug therapy.^16^ Data regarding the effect of amiodarone on EGM characteristics during VT ablation are limited. Amiodarone pre-VT ablation has been shown to temporarily mask LAVAs and LPs in ICM substrate, resulting in less extensive mapping and ablation times in amiodarone-treated compared to amiodarone-naïve patients.^17^ A higher VT recurrence rate was reported in amiodarone-treated patients upon amiodarone cessation. The study reported comparable low-voltage scar areas within the two groups. Contrasting findings have been reported in patients with A(R)VC despite similar scar areas, that is, amiodarone-naïve patients had smaller endocardial and epicardial areas with abnormal EGMs than amiodarone-treated patients.^18^ Consistently, however, long-term ablation outcomes were worse in the amiodarone-treated group.

In the present study, we provide the first report of the impact of chronic amiodarone therapy on omnipolar voltages and PF measurements. Amiodarone did not affect the low-voltage or the critical isthmus-measured areas. However, within the amiodarone-treated cohort, IZ PF was significantly lower in SR substrate maps (222 Hz vs. 303 Hz; *P* = 0.009) with similar non-significant trends observed in the RV paced and VT activation maps. This has relevant mapping implications as the optimal threshold to identify the IZ was higher at 220 Hz in the SR substrate maps from amiodarone-naïve patients compared with the total cohort (200 Hz). The effect of amiodarone on the IZ PF is an additional factor to consider. A recent analysis of PF specifically at VT termination sites during VT activation mapping reported an inverse correlation between PF and time to VT termination with RF.^20^ The average PF at the termination sites in ICM and NICM groups was 340 ± 116 and 402 ± 111 Hz, respectively. A high PF threshold of 405 Hz correlated with VT termination within 5 s after RF. Of the 69 patients, 31 were receiving amiodarone; however, analysis based on amiodarone therapy was not reported. In the present study, the IZ PF during VT activation mapping was significantly lower in the amiodarone-treated compared to amiodarone-naïve patients (172 Hz vs. 229 Hz; *P* = 0.002). It is therefore plausible that the optimal PF threshold for rapid VT termination may be lower in amiodarone-treated patients.

### Role of peak frequency in VT ablation

The current study provides an in-depth analysis of PF values obtained from 85 419 SR, 24 007 RV paced, and 42 391 VT activation map-derived EGMs. Combining PF with low-voltage substrate maps improves the diagnostic accuracy of low voltage-guided ablation; however, the performance metrics appear to be lower than with previously reported functional mapping techniques.

The concept of PF in VT substrate mapping is based on the observation that fractionated local EGMs are well-established markers for scarred ventricular myocardium, a necessary substrate for re-entrant VT circuits.^31–33^ In a canine pre-clinical post-MI model, fractionated EGMs were recorded in regions where infarct healing caused a wide separation of individual myocardial fibres while distorting fibre orientation.^31^ Fractionation was also shown to be independent of conduction slowing that is known to range between 0.6 and 1 ms^−1^ within disorganised fibres.^32^ Therefore, despite demonstrating a high correlation to manually verified fractionation, the lack of sensitivity to conduction slowing may explain the limitations of PF substrate mapping. Both ILAM and DeEP mapping methodologies have been shown to correlate with localized regions of conduction velocity slowing.^34,35^ Local PF values will additionally be dependent on catheter contact, pole orientation, and substrate depth within the tissue.

To ensure accurate timing characteristics, functional mapping methodologies, such as ILAM and DeEP, require correct annotation of the local NF as opposed to FF EGM components which are not functionally relevant. Furthermore, quantification of the transmural activation interval (TAI) in predicting intramural scar is dependent on accurate local timing annotation.^36^ The PF algorithm annotates the highest frequency component within a complex EGM. On the one hand, this identifies the NF component facilitating timing annotation which may improve the aforementioned mapping techniques. On the other hand, the frequency magnitude may provide the understanding of the proximity of the local NF signal to the mapping catheter, thus providing selective information at the mapped tissue plane. This is important as only a minority of re-entrant VT circuits are identifiable on the endocardial or epicardial surface alone.^6^ FF components may represent ventricular activation remote from the IZ, and however equally plausible, FF components may represent deeper intra-mural ventricular activation within a meandering three-dimensional (3D) isthmus. Therefore, a combination of relevant NF and FF signal annotation may provide a more accurate understanding of the complex 3D substrate and additionally enhance the performance of novel heart failure predictors that assess global viable myocardium.^37^ Clearly, pre-clinical studies providing a histological profile of the VT isthmus and/or human isthmus imaging studies (CT/MRI) with combined endocardial and epicardial mapping approaches would be required to substantiate this hypothesis.

Finally, the long-term benefits of extensive substrate ablation have been previously highlighted.^6^ We observed a trend towards a reduced number of ablation lesions and total RF time within the cohort of lesions above the 200 Hz low-voltage PF threshold. Higher PF MDPs have also been shown to correlate with rapid VT termination sites.^20^ Using substrate-based PF maps to guide ablation may therefore improve the efficiency of the procedure by focusing ablation energy to responsive substrate.

### Study limitations

This is a mechanistic study based on a retrospective offline analysis of a cohort of 30 patients exploring the role of substrate-based PF mapping. Reported procedural outcomes were non-randomised and based on local workflows in high-volume centres. Substrate mapping was performed in SR or RV pacing with no available data for biventricular or LV only substrate maps. The wavelet-transform OTNF algorithm operates only on peak-to-peak voltage amplitudes of ≥0.04 mV, which aims to prevent errors due to low-energy, high-frequency noise. However, very high-frequency noise with voltage amplitude ≥0.04 mV may have been included within the analysis. Only high-density maps were included within the final analysis to mitigate against poor electrode-tissue contact that may result in lower-frequency EGMs; however, this cannot be guaranteed. Low-frequency NF EGMs of clinical significance may reflect critical intramural substrate; however, the value of PF mapping may be in the identification of substrate amenable to catheter ablation. We also acknowledge that, as part of the study design to assess for diagnostic accuracy, only mappable VTs were included within the analysis.

### Future perspectives

The ability of PF-guided low-voltage substrate mapping to improve VT ablation procedural and RF metrics should be tested in the setting of a randomised controlled trial. Furthermore, combining the OTNF algorithm with ILAM, DeEP, and TAI methods may improve the performance of these substrate mapping techniques and provide further insights into the complex 3D structure of the critical isthmus.

## Conclusions

PF can be combined with low-voltage substrate maps to highlight critical components of the VT re-entry circuit. Isthmus PF is influenced by chronic amiodarone therapy with lower values observed during RV pacing, with an optimal diagnostic accuracy using a threshold of ≥200 Hz during SR substrate mapping.

## Supplementary Material

euae253_Supplementary_Data

## Data Availability

The data underlying this article will be shared on reasonable request to the corresponding author.

## References

[1] Vergara P, Trevisi N, Ricco A, Petracca F, Baratto F, Cireddu M et al Late potentials abolition as an additional technique for reduction of arrhythmia recurrence in scar related ventricular tachycardia ablation. J Cardiovasc Electrophysiol 2012;23:621–7. 22486970 10.1111/j.1540-8167.2011.02246.x

[2] Sacher F, Lim HS, Derval N, Denis A, Berte B, Yamashita S et al Substrate mapping and ablation for ventricular tachycardia: the LAVA approach. J Cardiovasc Electrophysiol 2015;26:464–71. 25328104 10.1111/jce.12565

[3] Tzou WS, Frankel DS, Hegeman T, Supple GE, Garcia FC, Santangeli P et al Core isolation of critical arrhythmia elements for treatment of multiple scar-based ventricular tachycardias. Circ Arrhythm Electrophysiol 2015;8:353–61. 25681389 10.1161/CIRCEP.114.002310

[4] Berruezo A, Fernandez-Armenta J, Andreu D, Penela D, Herczku C, Evertz R et al Scar dechanneling: new method for scar-related left ventricular tachycardia substrate ablation. Circ Arrhythm Electrophysiol 2015;8:326–36. 25583983 10.1161/CIRCEP.114.002386

[5] Rossi P, Cauti FM, Niscola M, Magnocavallo M, Polselli M, Capone S et al Ventricular electrograms duration map to detect ventricular arrhythmia substrate: the VEDUM project study. Circ Arrhythm Electrophysiol 2023;16:447–55. 37485678 10.1161/CIRCEP.122.011729 PMC10786440

[6] Natale A, Zeppenfeld K, Della Bella P, Liu X, Sabbag A, Santangeli P et al Twenty-five years of catheter ablation of ventricular tachycardia: a look back and a look forward. Europace 2023;25:euad225. 37622589 10.1093/europace/euad225 PMC10451002

[7] Aziz Z, Shatz D, Raiman M, Upadhyay GA, Beaser AD, Besser SA et al Targeted ablation of ventricular tachycardia guided by wavefront discontinuities during Sinus rhythm: a new functional substrate mapping strategy. Circulation 2019;140:1383–97. 31533463 10.1161/CIRCULATIONAHA.119.042423

[8] Al-Sheikhli J, Winter J, Luque IR, Lambiase PD, Orini M, Porta-Sanchez A et al Optimization of decrementing evoked potential mapping for functional substrate identification in ischaemic ventricular tachycardia ablation. Europace 2023;25:euad092. 37032650 10.1093/europace/euad092 PMC10228600

[9] Porta-Sanchez A, Jackson N, Lukac P, Kristiansen SB, Nielsen JM, Gizurarson S et al Multicenter study of ischemic ventricular tachycardia ablation with decrement-evoked potential (DEEP) mapping with extra stimulus. JACC Clin Electrophysiol 2018;4:307–15. 30089555 10.1016/j.jacep.2017.12.005

[10] Guichard JB, Regany-Closa M, Vazquez-Calvo S, Zazu B, Pellicer Sendra B, Serrano-Campaner J et al Substrate mapping for ventricular tachycardia ablation through high-density whole-chamber double extra stimuli: the S3 protocol. JACC Clin Electrophysiol 2024;10:1534–47. 38819348 10.1016/j.jacep.2024.04.023

[11] Irie T, Yu R, Bradfield JS, Vaseghi M, Buch EF, Ajijola O et al Relationship between sinus rhythm late activation zones and critical sites for scar-related ventricular tachycardia: systematic analysis of isochronal late activation mapping. Circ Arrhythm Electrophysiol 2015;8:390–9. 25740836 10.1161/CIRCEP.114.002637 PMC4695215

[12] Berte B, Relan J, Sacher F, Pillois X, Appetiti A, Yamashita S et al Impact of electrode type on mapping of scar-related VT. J Cardiovasc Electrophysiol 2015;26:1213–23. 26198475 10.1111/jce.12761

[13] Okubo K, Frontera A, Bisceglia C, Paglino G, Radinovic A, Foppoli L et al Grid mapping catheter for ventricular tachycardia ablation. Circ Arrhythm Electrophysiol 2019;12:e007500. 31500436 10.1161/CIRCEP.119.007500

[14] Proietti R, Dowd R, Gee LV, Yusuf S, Panikker S, Hayat S et al Impact of a high-density grid catheter on long-term outcomes for structural heart disease ventricular tachycardia ablation. J Interv Card Electrophysiol 2021;62:519–29. 33392856 10.1007/s10840-020-00918-4 PMC8645535

[15] Zimetbaum P . Antiarrhythmic drug therapy for atrial fibrillation. Circulation 2012;125:381–9. 22249528 10.1161/CIRCULATIONAHA.111.019927

[16] Zipes DP, Prystowsky EN, Heger JJ. Amiodarone: electrophysiologic actions, pharmacokinetics and clinical effects. J Am Coll Cardiol 1984;3:1059–71. 6368644 10.1016/s0735-1097(84)80367-8

[17] Di Biase L, Romero J, Du X, Mohanty S, Trivedi C, Della Rocca DG et al Catheter ablation of ventricular tachycardia in ischemic cardiomyopathy: impact of concomitant amiodarone therapy on short- and long-term clinical outcomes. Heart Rhythm 2021;18:885–93. 33592323 10.1016/j.hrthm.2021.02.010

[18] Lin CY, Chung FP, Nwe N, Hsieh YC, Li CH, Lin YJ et al Impact of amiodarone therapy on the ablation outcome of ventricular tachycardia in arrhythmogenic right ventricular cardiomyopathy. J Clin Med 2022;11:7265. 36555882 10.3390/jcm11247265 PMC9787968

[19] Payne JE, Woods C, Elshazly MB, Matthews A, Kroman A, Feng Z et al A novel automated peak frequency annotation algorithm for identifying deceleration zones and ventricular tachycardia ablation sites. Heart Rhythm 2024;21:27–33. 37852563 10.1016/j.hrthm.2023.10.014

[20] Cauti FM, Martini N, Fioravanti F, Tanese N, Magnocavallo M, Rampa L, et al Analysis of electrograms peak frequency during ventricular tachycardia ablation. Insights into human tridimensional ventricular tachycardia circuits. Heart Rhythm 2024:S1547–5271(24)02697-3. doi:10.1016/j.hrthm.2024.06.014 38880202

[21] Quinto L, Sanchez-Somonte P, Alarcon F, Garre P, Castillo A, San Antonio R et al Ventricular tachycardia burden reduction after substrate ablation: predictors of recurrence. Heart Rhythm 2021;18:896–904. 33639298 10.1016/j.hrthm.2021.02.016

[22] Julia J, Bokhari F, Uuetoa H, Derejko P, Traykov VB, Gwizdala A et al A new era in epicardial access for the ablation of ventricular arrhythmias: the Epi-Co(2) registry. JACC Clin Electrophysiol 2021;7:85–96. 33478716 10.1016/j.jacep.2020.07.027

[23] Al-Sheikhli J, Patchett I, Lim VG, Marshall L, Foster W, Kuehl M et al Initial experience of temperature-controlled irrigated radiofrequency ablation for ischaemic cardiomyopathy ventricular tachycardia ablation. J Interv Card Electrophysiol 2023;66:551–9. 35192098 10.1007/s10840-022-01158-4 PMC10066113

[24] Dhanjal T, Tondo C, Anselme F, Jacobs S, Burrell J, Becker D et al Safety and efficacy of a temperature-controlled ablation system for ventricular tachycardia: first results from the TRAC-VT study. Europace 2024;26:euae102–780. 10.1007/s10840-025-01995-z PMC12399702 39893296

[25] Hsia HH, Lin D, Sauer WH, Callans DJ, Marchlinski FE. Anatomic characterization of endocardial substrate for hemodynamically stable reentrant ventricular tachycardia: identification of endocardial conducting channels. Heart Rhythm 2006;3:503–12. 16648052 10.1016/j.hrthm.2006.01.015

[26] Verma A, Marrouche NF, Schweikert RA, Saliba W, Wazni O, Cummings J et al Relationship between successful ablation sites and the scar border zone defined by substrate mapping for ventricular tachycardia post-myocardial infarction. J Cardiovasc Electrophysiol 2005;16:465–71. 15877614 10.1046/j.1540-8167.2005.40443.x

[27] Mountantonakis SE, Park RE, Frankel DS, Hutchinson MD, Dixit S, Cooper J et al Relationship between voltage map “channels” and the location of critical isthmus sites in patients with post-infarction cardiomyopathy and ventricular tachycardia. J Am Coll Cardiol 2013;61:2088–95. 23524215 10.1016/j.jacc.2013.02.031

[28] Hsia HH, Callans DJ, Marchlinski FE. Characterization of endocardial electrophysiological substrate in patients with nonischemic cardiomyopathy and monomorphic ventricular tachycardia. Circulation 2003;108:704–10. 12885746 10.1161/01.CIR.0000083725.72693.EA

[29] Srinivasan NT, Garcia J, Schilling RJ, Ahsan S, Babu GG, Ang R et al Multicenter study of dynamic high-density functional substrate mapping improves identification of substrate targets for ischemic ventricular tachycardia ablation. JACC Clin Electrophysiol 2020;6:1783–93. 33357574 10.1016/j.jacep.2020.06.037 PMC7769061

[30] Beheshti M, Nayyar S, Magtibay K, Masse S, Porta-Sanchez A, Haldar S et al Quantifying the determinants of decremental response in critical ventricular tachycardia substrate. Comput Biol Med 2018;102:260–6. 29871758 10.1016/j.compbiomed.2018.05.025

[31] Gardner PI, Ursell PC, Fenoglio JJ Jr, Wit AL. Electrophysiologic and anatomic basis for fractionated electrograms recorded from healed myocardial infarcts. Circulation 1985;72:596–611. 4017211 10.1161/01.cir.72.3.596

[32] de Bakker JM, van Capelle FJ, Janse MJ, Tasseron S, Vermeulen JT, de Jonge N et al Slow conduction in the infarcted human heart. ‘Zigzag’ course of activation. Circulation 1993;88:915–26. 8353918 10.1161/01.cir.88.3.915

[33] Joy G, Lopes LR, Webber M, Ardissino AM, Wilson J, Chan F et al Electrophysiological characterization of subclinical and overt hypertrophic cardiomyopathy by magnetic resonance imaging-guided electrocardiography. J Am Coll Cardiol 2024;83:1042–55. 38385929 10.1016/j.jacc.2024.01.006 PMC10945386

[34] Raiman M, Tung R. Automated isochronal late activation mapping to identify deceleration zones: rationale and methodology of a practical electroanatomic mapping approach for ventricular tachycardia ablation. Comput Biol Med 2018;102:336–40. 30033360 10.1016/j.compbiomed.2018.07.012

[35] Jackson N, Gizurarson S, Viswanathan K, King B, Masse S, Kusha M et al Decrement evoked potential mapping: basis of a mechanistic strategy for ventricular tachycardia ablation. Circ Arrhythm Electrophysiol 2015;8:1433–42. 26480929 10.1161/CIRCEP.115.003083

[36] Venlet J, Piers SR, Hoogendoorn J, Androulakis AFA, de Riva M, van der Geest RJ et al The transmural activation interval: a new mapping tool to identify ventricular tachycardia substrates in right ventricular cardiomyopathy. Europace 2023;25:478–86. 36480385 10.1093/europace/euac220 PMC9935041

[37] Rademaker R, Kimura Y, de R, Silva M, Beukers HC, Piers SRD et al Area-weighted unipolar voltage to predict heart failure outcomes in patients with ischaemic cardiomyopathy and ventricular tachycardia. Europace 2024;26:euad346. 38308809 10.1093/europace/euad346 PMC10838146

