# Correction to: Detailed analysis of electrogram peak frequency to guide ventricular tachycardia substrate mapping

**DOI:** 10.1093/europace/euae303

**Published:** 2024-12-30

This is a correction to: Joseph Mayer, Jaffar Al-Sheikhli, Maria Niespialowska-Steuden, Ian Patchett, James Winter, Rafaella Siang, Nicolas Lellouche, Karthick Manoharan, Thanh Trung Phan, Justo Juliá Calvo, Andreu Porta-Sánchez, Ivo Roca-Luque, John Silberbauer, Tarvinder Dhanjal, Detailed analysis of electrogram peak frequency to guide ventricular tachycardia substrate mapping, *EP Europace*, Volume 26, Issue 10, October 2024, euae253, https://doi.org/10.1093/europace/euae253

In the originally published version of this manuscript, author Ivo Roca-Luque was incorrectly listed as Ivo Roca Luque.

This error has been corrected.

