# Supplementary material for: Detailed analysis of electrogram peak frequency to guide ventricular tachycardia substrate mapping
Source: Europace. 2024 Sep 29;26(10):euae253. doi: 10.1093/europace/euae253 (PMC11481296; doi:10.1093/europace/euae253)
Supplement: euae253_Supplementary_Data [file euae253_supplementary_data.zip › Supplementary Table 1.docx]

Supplementary Table 1: Sinus Rhythm substrate, Right Ventricular paced substrate and VT activation map characteristics.

|  | | | | p-value | | | |
| --- | --- | --- | --- | --- | --- | --- | --- |
|  | SR substrate map | RVp substrate map | VT activation map | global | SR-RVp | SR-VT | RVp-VT |
| Number of maps | 30 | 21 | 32 |  |  |  |  |
| Total map points used, n (IQR) | 3531 (2599-5228) | 1224 (682-1820) | 1487 (788-2791) | **<0.001** | **<0.001** | **<0.001** | 0.27 |
| Total map area, cm^2^ | 198.0±63.1 | 169.6±55.0 | 131.5±70.7 | **0.001** | 0.09 | **0.001** | **0.042** |
| Total map PF, Hz (IQR) | 205 (184-222) | 191 (IQR: 164-214) | 202 (IQR: 178-232) | 0.15 | - | - | - |
| Total map voltage, mV | 0.74±0.33 | 0.81±0.51 | 0.39±0.54 | 0.12 | - | - | - |
| Map area >1.5mV PF, Hz (IQR) | 269 (247-291) | 251 (219-282) | 252 (217-287) | 0.27 | - | - | - |
| Low voltage (<1.5mV) area, cm^2^ | 131.8±48.9 | 118.4±44.9 | 97.93±65.2 | **0.003** | 0.38 | **0.001** | **0.021** |
| Low voltage (<1.5mV) area PF, Hz (IQR) | 173 (158-191) | 162 (148-190) | 177 (165-220) | 0.05 | - | - | - |
| VT Isthmus area, cm^2^ | 12.33±7.84 | 12.33±7.84 | 12.33±7.84 | - | - | - | - |
| VT Isthmus area PF, Hz (IQR) | 234 (195-294) | 197 (166-220) | 220 (193-294) | **0.025** | **0.010** | 0.06 | **0.022** |
| VT Isthmus area voltage, mV | 0.52±0.45 | 0.62±0.82 | 0.34±0.26 | **0.035** | 0.96 | **0.023** | **0.031** |

If a global statistical difference between the different maps was found (global p<0.05), a detailed analysis was performed among the different groups. P values in bold denote statistical significance (p<0.05). **SR**: Sinus Rhythm; **RVp**: Right Ventricular paced; **VT**: Ventricular Tachycardia; **IQR:** Interquartile range; **PF:** Peak Frequency; **Hz:** Hertz; **mV:** Millivolts.

There were no significant differences across the three map types in terms of total map PF, total map omnipolar voltage, PF in the LVA (<1.5mV) and PF in healthy myocardium (>1.5mV). Within the IZ, significant differences were seen in omnipolar voltage (p=0.035) with VT activation map voltages lower than SR substrate map (p=0.023) and RVp substrate map (p=0.031) voltages. There were no differences between the SR and RVp substrate map IZ voltages.
