# Supplementary material for: Detailed analysis of electrogram peak frequency to guide ventricular tachycardia substrate mapping
Source: Europace. 2024 Sep 29;26(10):euae253. doi: 10.1093/europace/euae253 (PMC11481296; doi:10.1093/europace/euae253)
Supplement: euae253_Supplementary_Data [file euae253_supplementary_data.zip › Supplementary Table 2.docx]

|  |  |  |  | p-value | | | |
| --- | --- | --- | --- | --- | --- | --- | --- |
|  | SR map | RVp map | VT Activation Map | global | SR-RVp | SR-VT | RVp-VT |
| Number of maps | 21 | 21 | 21 |  | | | |
| Total map points used, n (IQR) | 3537 (2658-4500) | 1224 (682-1820) | 1134(698-2467) | **<0.001** | <0.001 | <0.001 | 0.83 |
| Total map area, cm^2^ | 200.0±58.3 | 169.6±55 | 133.0±79.0 | **0.002** | 0.06 | **0.002** | **0.040** |
| Total map PF, Hz (IQR) | 201 (181-230) | 191 (164-214) | 199 (178-234) | 0.20 | - | - | - |
| Total map voltage, mV | 0.73±0.34 | 0.81±0.51 | 0.62±0.56 | 0.16 | - | - | - |
| Map area >1.5mV PF, Hz (IQR) | 281(252-292) | 251 (219-282) | 255 (220-316) | 0.20 | **-** | - | - |
| Low voltage (<1.5mV) area, cm^2^ | 127.1±41.3 | 118.4±44.9 | 94.6±69.1 | **0.003** | 0.17 | **0.001** | **0.020** |
| Low voltage (<1.5mV) area PF, Hz (IQR) | 173 (153-198) | 162 (148-190) | 174 (165-217) | 0.10 | - | - | **-** |
| VT Isthmus area, cm^2^ | 11.9±5.32 | 11.9±5.32 | 11.9±5.32 | - | - | - | - |
| VT Isthmus area PF, Hz (IQR) | 244 (201-312) | 197 (166-220) | 220 (189-279) | **0.010** | **0.003** | 0.26 | 0.06 |
| VT Isthmus area voltage, mV | 0.55±0.51 | 0.62±0.82 | 0.35±0.3 | 0.07 | - | **-** | - |

Supplementary Table 2: SR substrate, RV paced substrate and VT activation map characteristics within cohort of patient-matched maps.

**SR**: Sinus Rhythm; **RVp**: Right Ventricular paced; **VT**: Ventricular Tachycardia; **IQR:** Interquartile range; **PF:** Peak Frequency; **Hz:** Hertz; **mV:** Millivolts.
