# Supplementary figures and images for: Detailed analysis of electrogram peak frequency to guide ventricular tachycardia substrate mapping
Source: Europace. 2024 Sep 29;26(10):euae253. doi: 10.1093/europace/euae253 (PMC11481296; doi:10.1093/europace/euae253)

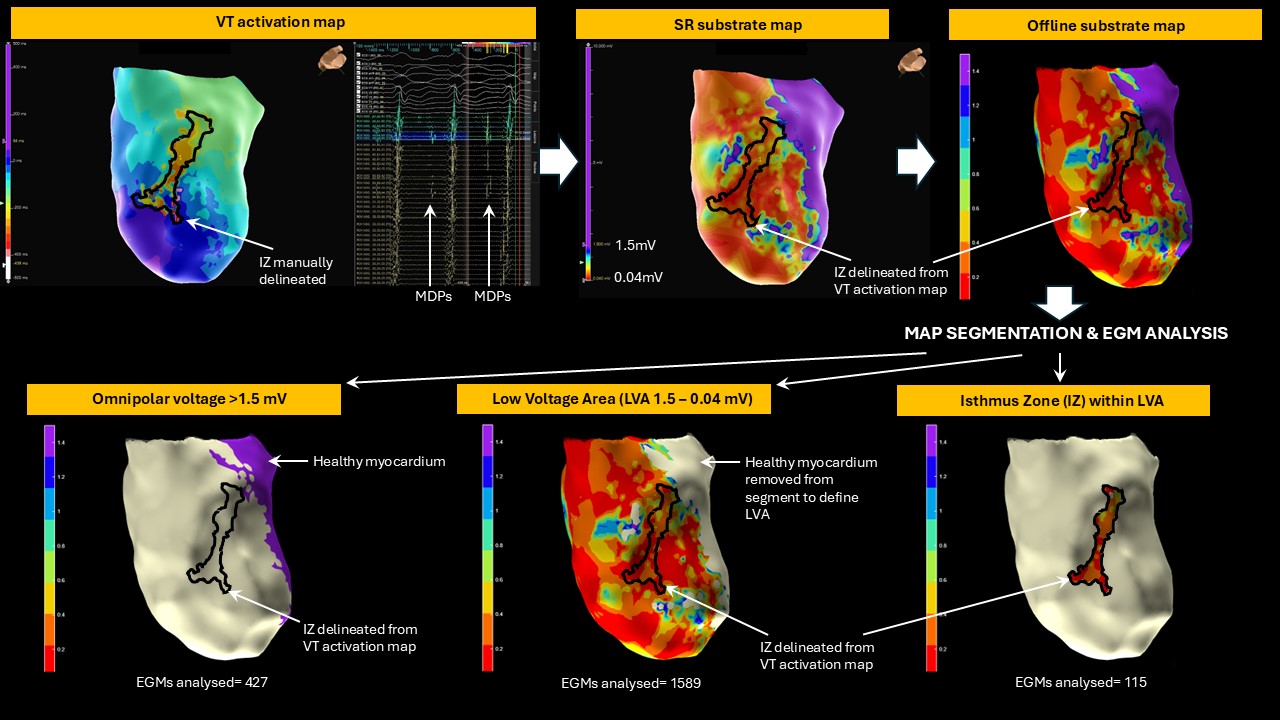

Supplement: euae253_Supplementary_Data [file euae253_supplementary_data.zip › Supplementary Figure 1.jpg]

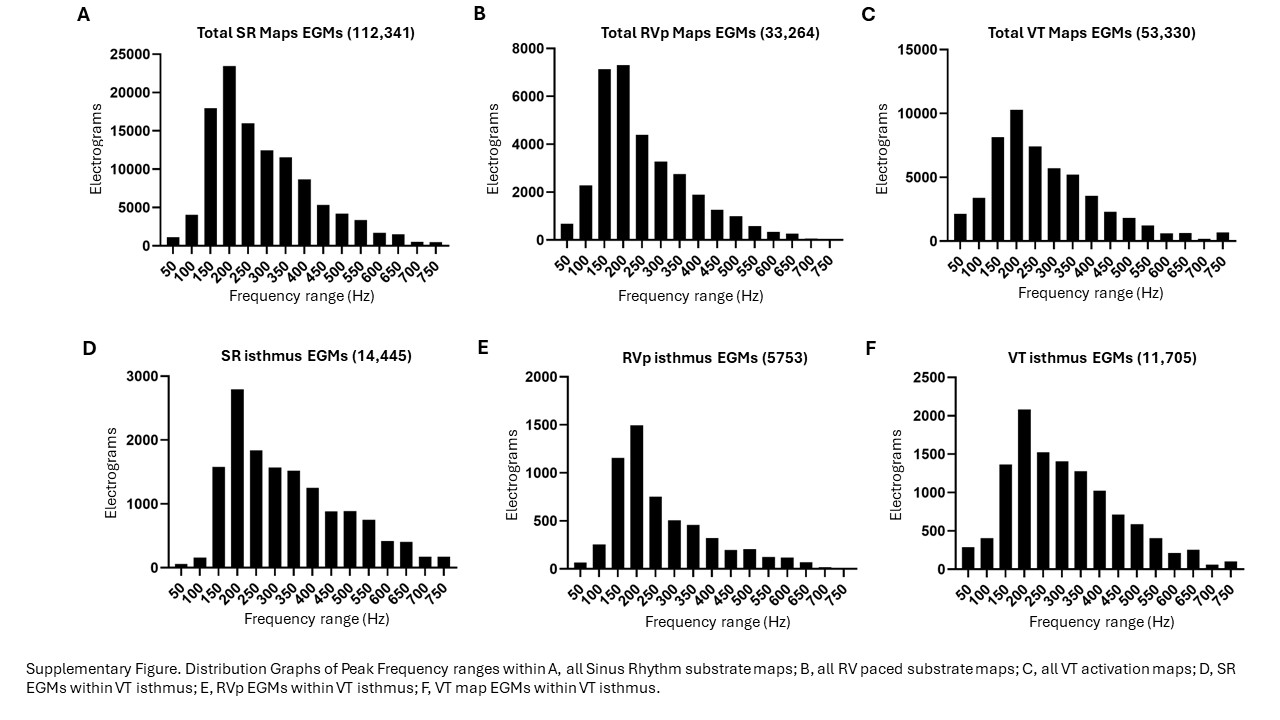

Supplement: euae253_Supplementary_Data [file euae253_supplementary_data.zip › Supplementary Figure 2.jpg]
